# The characteristic patterns of individual brain susceptibility networks underlie Alzheimer’s disease and white matter hyperintensity-related cognitive impairment

**DOI:** 10.1038/s41398-024-02861-8

**Published:** 2024-04-04

**Authors:** Haifeng Chen, Jingxian Xu, Weikai Li, Zheqi Hu, Zhihong Ke, Ruomeng Qin, Yun Xu

**Affiliations:** 1 Nanjing Drum Tower Hospital Clinical College of Traditional Chinese and Western Medicine, Nanjing University of Chinese Medicine, Nanjing, China; 2 Department of Neurology, Nanjing Drum Tower Hospital, Affiliated Hospital of Medical School, Nanjing University, Nanjing, China; 3 Jiangsu Key Laboratory of Molecular Medicine, Medical School of Nanjing University, Nanjing, China; 4 Jiangsu Province Stroke Center for Diagnosis and Therapy, Nanjing, China; 5 Nanjing Neuropsychiatry Clinic Medical Center, Nanjing, China; 6 School of Mathematics and Statistics, Chongqing Jiaotong University, Chongqing, China; 7 MIIT Key Laboratory of Pattern Analysis and Machine Intelligence, Nanjing, China

**Keywords:** Long-term memory, Hippocampus

## Abstract

Excessive iron accumulation in the brain cortex increases the risk of cognitive deterioration. However, interregional relationships (defined as susceptibility connectivity) of local brain iron have not been explored, which could provide new insights into the underlying mechanisms of cognitive decline. Seventy-six healthy controls (HC), 58 participants with mild cognitive impairment due to probable Alzheimer’s disease (MCI-AD) and 66 participants with white matter hyperintensity (WMH) were included. We proposed a novel approach to construct a brain susceptibility network by using Kullback‒Leibler divergence similarity estimation from quantitative susceptibility mapping and further evaluated its topological organization. Moreover, sparse logistic regression (SLR) was applied to classify MCI-AD from HC and WMH with normal cognition (WMH-NC) from WMH with MCI (WMH-MCI).The altered susceptibility connectivity in the MCI-AD patients indicated that relatively more connectivity was involved in the default mode network (DMN)-related and visual network (VN)-related connectivity, while more altered DMN-related and subcortical network (SN)-related connectivity was found in the WMH-MCI patients. For the HC vs. MCI-AD classification, the features selected by the SLR were primarily distributed throughout the DMN-related and VN-related connectivity (accuracy = 76.12%). For the WMH-NC vs. WMH-MCI classification, the features with high appearance frequency were involved in SN-related and DMN-related connectivity (accuracy = 84.85%). The shared and specific patterns of the susceptibility network identified in both MCI-AD and WMH-MCI may provide a potential diagnostic biomarker for cognitive impairment, which could enhance the understanding of the relationships between brain iron burden and cognitive decline from a network perspective.

## Introduction

Iron plays a central role in many basic biological processes in the central nervous system, such as oxidative phosphorylation, neurotransmission and myelin synthesis [1]. Emerging evidence suggests that excessive iron accumulation in cortical and subcortical gray matter (GM) increases the risk of cognitive deterioration [2, 3]. Previous studies have focused on local brain iron deposition with region-based approaches [2, 3]. However, interregional relationships (referred to as susceptibility connectivity) of local brain iron in individuals have not been explored, which might provide additional information for further understanding the neuromechanism of cognitive decline.

Quantitative susceptibility mapping (QSM) is a validated and noninvasive technique for detecting brain iron concentrations in vivo via tissue susceptibility values [4, 5]. The regulation of iron homoeostasis is crucial to normal brain function, whereas dysregulation can lead to neurotoxicity through different mechanisms. During ageing, abnormal iron burden occurs in specific brain regions, including the basal ganglia, hippocampus, and other subcortical brain regions related to cognitive impairment [6]. Several studies on QSM have reported elevated brain magnetic susceptibility in Alzheimer’s disease (AD) [2, 7, 8]. Across the spectrum of AD with positive amyloid-β (Aβ) load, higher susceptibility in the hippocampal cortex could predict accelerated deterioration of episodic memory, executive function, and attention function [8]. This finding indicated that brain iron might bind to Aβ to accelerate clinical progression. In contrast to those in AD, the presence and pattern of iron accumulation in vascular cognitive impairment (VCI) are less consistent. Using QSM, Moon et al. proposed that individuals with VCI exhibit elevated iron accumulation in the caudate and putamen nuclei [9]. However, another study reported no differences in the iron content in the postmortem brains of patients with VCI compared with that in the brains of healthy elderly individuals [10]. Previous brain iron-related studies have mainly concentrated on iron deposition via region-based approaches. Indeed, the brain functions as a complex system that allows information to be segregated and integrated efficiently at low wiring costs. Recent studies have modeled the brain as complex networks that are linked by structural connectivity (i.e., constructed by diffusion tensor imaging [DTI] or 3D T_1_ imaging), functional connectivity (i.e., constructed by rs-fMRI) or metabolic connectivity (i.e., constructed by positron emission tomography [PET]). To date, the concept of the individual “susceptibility network” based on QSM data has not been mentioned, which may provide a more comprehensive understanding of the metabolic function of the brain from a network perspective.

Inspired by morphological brain networks based on Kullback‒Leibler divergence similarity estimation (KLSE) [11–13], we developed a new analytic method for constructing an individual-level susceptibility network related to brain iron deposition using QSM imaging. Technically, brain iron levels can be effectively determined from susceptibility values in a specific brain region via QSM images. KLSE could quantify the interregional relationships (i.e., susceptibility connectivity) for a single participant by estimating the similarity of brain iron distributions. We suppose that the relatively high similarity of brain iron distributions in any two regions reflects relatively more interregional information transmission. This framework provides a potential approach to explore intra- and inter-individual differences in susceptibility networks in individuals with cognitive disorders.

In this study, our team used KLSE to construct a brain susceptibility network at the individual level from QSM data and further evaluated its reproducibility and reliability. Moreover, a graph-based analysis was used to explore and compare the pattern and presence of abnormal connectivity and topological organization of the susceptibility network between AD-related and vascular-related cognitive impairment. Finally, we combined machine learning methods with the susceptibility network to assess the classification performance of disease diagnosis. This study may offer the opportunity to further understand the mechanisms underlying different types of cognitive impairment and provide a potential diagnostic biomarker from the perspective of susceptibility networks.

## Materials and methods

### Participants

This cross-sectional study was approved by the Ethics Committee of Nanjing Drum Tower Hospital, and informed consent was obtained from all participants (clinical trial registration number: ChicTR-00C-17010562). After checking the data integrity of the cognitive assessments and multimodal MRI, 76 healthy controls (HC), 58 patients with mild cognitive impairment due to probable Alzheimer’s disease (MCI-AD), and 66 participants with moderate to severe WMH (aged 50–80 years) were included. Participants with moderate to severe WMH were categorized as WMH with normal cognition (WMH-NC, *n* = 31) and WMH with MCI (WMH-MCI, *n* = 35) based on cognitive evaluation. The diagnostic criteria of cognitive impairment was described in the Supplementary Materials. Moderate to severe WMH were identified according to WMH Fazekas scale 2 or 3. The MCI-AD individuals were diagnosed according to the recommendations of Petersen et al. [14] and the National Institute on Aging-Alzheimer’s Association [15]. Notably, the MCI-AD individuals with moderate to severe WMH were excluded, with the aim of minimizing the possibility of mixed dementia. The exclusion criteria included multiple cerebral infarctions, multiple cerebral microbleeds, nonvascular WMH, and other neurological/psychiatric diseases.

### Cognitive function measurement

All participants underwent a standardized neuropsychological examination. General cognitive performance was evaluated by the Mini-Mental State Examination (MMSE) and the Beijing version of the Montreal Cognitive Assessment (MoCA-BJ). Multiple cognitive domain tests included memory function, visuospatial function, language function, executive function and information processing speed, which are described in the Supplementary Materials.

### MRI scanning

The multimodal neuroimaging data were acquired using a Philips Medical Systems 3.0 T machine. The protocol included high-resolution 3D T_1_ imaging, 3D fluid-attenuated inversion recovery (FLAIR) imaging and 3D fast field-echo imaging. The detailed scanning sequence is described in the Supplementary Materials.

### Segmentation and volume determination

WMH segmentation was conducted based on the lesion prediction algorithm in the Lesion Segmentation Tool (LST 2.0.15; https://www.statistical-modelling.de/lst.html) [16]. The extracted lesion probability map of each individual was visually inspected against the corresponding FLAIR image.

Brain tissue segmentation was performed with the Computational Anatomy Toolbox (http://dbm.neuro.uni-jena.de/cat/). The main processing steps included correction for bias-field inhomogeneities, spatial normalization with Diffeomorphic Anatomical Registration using the Exponentiated Lie algebra (DARTEL) algorithm and tissue segmentation into white matter (WM), GM and cerebrospinal fluid (CSF). The total intracranial volume (TIV) was obtained by summing the volumes of the GM, WM and CSF. To normalize the head size of each individual, we defined the normalized GM volume (GMV) as the ratio of GMV divided by TIV (GMV/TIV ratio). Due to the potential effect of GMV on the susceptibility value, we assessed the GMV/TIV in each participant.

### Susceptibility map reconstruction and normalization

The individual susceptibility map was reconstructed from the magnitude and phase images using the STI Suite (https://people.eecs.berkeley.edu/~chunlei.liu/software.html). First, a single-subject brain mask was extracted from the magnitude image. Second, phase unwrapping was conducted by using a Laplacian-based phase unwrapping algorithm [17]. Third, the variable-kernel sophisticated harmonic artifact reduction for phase data algorithm was applied to remove the background field from the phase images [18]. Finally, an individual susceptibility map was obtained using the streaking artefact reduction algorithm to calculate dipole inversion and reduce streaking artefacts [19].

The Statistical Parametric Mapping analysis package (SPM12, http://www.fil.ion.ucl.ac.uk/spm/software/spm12/) was used to normalize the individual susceptibility map to standard MNI space [20, 21]. First, a magnitude image acquired from 3D fast field-echo imaging was coregistered to the 3D T_1_ image. The susceptibility map was transformed based on the coregistration between the magnitude image and the 3D T_1_ image. Second, the coregistered susceptibility map was spatially normalized to the MNI space using the normalized transformation matrix of the 3D T_1_ image. Then, the spatially normalized susceptibility map was smoothed with the Gaussian kernel (8 mm full width at half-maximum). Finally, the smoothed susceptibility map was multiplied by the GM mask generated from GM segmentation processing (using the DARTEL algorithm) to obtain the individual susceptibility map of the GM for subsequent susceptibility network analysis.

### Susceptibility network construction

**Fig. 1:**
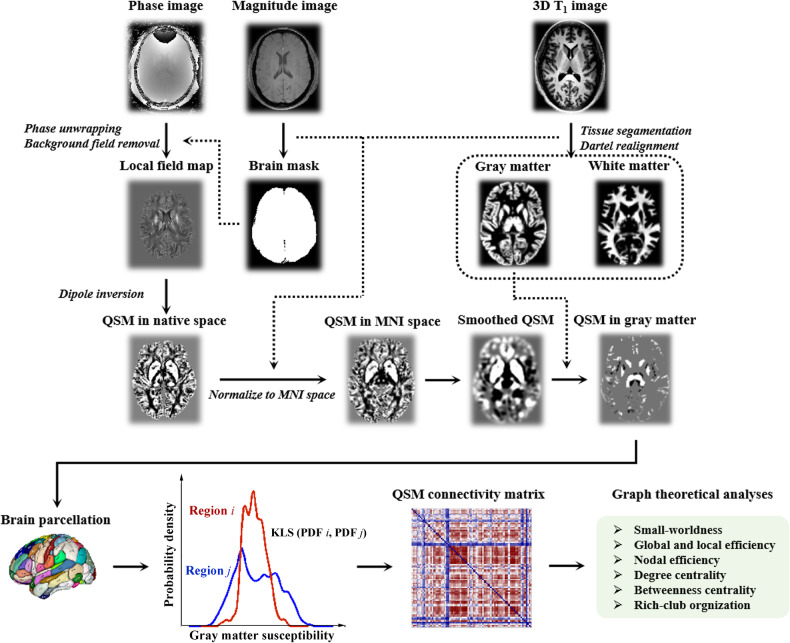
A flowchart illustrating the main construction process of the susceptibility network. QSM quantitative susceptibility mapping, MNI Montreal Neurological Institute, KLS Kullback-Leibler divergence similarity, PDF probability density function.

In this study, the similarity of magnetic susceptibility in any two brain regions from QSM images based on Kullback‒Leibler divergence was used to delineate individual susceptibility connections [11, 12]. By estimating the similarity of iron distributions, this framework is able to quantify the susceptibility connectivity for a single participant. First, we extracted the susceptibility values of each region of interest (ROI), which were applied to estimate the probability density function (PDF) via nonparametric kernel density estimation (KDE). This KDE analysis was conducted based on a public MATLAB script (http://www.mathworks.com/matlabcentral/fileexchange/14034-kernel-density-estimator). Second, the symmetric Kullback‒Leibler divergence based on the PDFs was computed according to the following mathematical equation:

1 $${D}_{{KL}}\left(I,J\right)=\mathop{\int }\nolimits_{X}\,(I\left(x\right)\log \frac{I\left(x\right)}{J\left(x\right)}+J(x)\log \frac{J(x)}{I(x)})$$

where *I* and *J* are the two PDFs of ROI_*i*_ and ROI_*j*_. Subsequently, the KLSE (i.e., the susceptibility connectivity) between ROI_*i*_ and ROI_*j*_ is computed by the Kullback‒Leibler divergence as follows:

2 $${KLSE}(I,J)={e}^{-{D}_{{KL}}(I,J)}$$

where *e* is the nature exponential. The KLSE ranges from 0 to 1, where 1 is for two identical distributions. Two parcellation schemes, the Anatomical Automatic Labelling atlas (AAL) and the Brainnetome atlas (BNA), were used to define the nodes (Supplementary Tables 1 and 2) [22]. Finally, KLSE-based 90 × 90 and 246 × 246 weighted susceptibility matrices were constructed for each subject. In addition to the weighted susceptibility network, we constructed a binary network derived by sparsity thresholds (from 0.05 to 0.4, interval = 0.01) to assess the stability of our findings [23]. A flowchart describing the main construction process of the susceptibility network is shown in Fig. 1.

### Susceptibility network analysis

#### Topological properties of the susceptibility network

We applied the Graph Theoretical Network Analysis Toolbox (GRETNA, http://www.nitrc.org/projects/gretna/) to analyze the topological properties of the susceptibility network [24]. The global network properties included small-world metrics [clustering coefficient (*C*_*p*_), normalized clustering coefficient (*γ*, Gamma), characteristic path length (*L*_*p*_), normalized characteristic path length (*λ*, Lambda) and small-worldness (*σ*, Sigma)] and network efficiency metrics [global efficiency (*E*_*glob*_) and local efficiency (*E*_*loc*_)]. In addition, betweenness centrality, degree centrality, nodal global efficiency and nodal local efficiency were used to evaluate the regional properties. Their definitions and mathematical equations have been described in previous studies, and we also mentioned this in Supplementary Table 3 [25, 26]. A network is regarded as small-worldness (i.e., *σ* = *γ*/*λ*) if it meets the following criterion: *σ* > 1 [27]. Moreover, we computed the area under the curve (AUC) over a range of sparsities for each network metric [23].

#### Rich-club organization analysis

The characteristics of rich-club organization in terms of both binary and weighted susceptibility networks were evaluated. To identify the existence of significant rich-club organization, the rich-club coefficient *φ*(*k*) and the weighted rich-club coefficient *φ*^*w*^(*k*) were normalized relative to a set of 1000 random networks. By definition, the normalized *φ*(*k*) and *φ*^*w*^(*k*) [*φ*_norm_(*k*) and *φ*^*w*^_norm_(*k*)] > 1 over a range of *k* are indicative of a rich-club organization within a network [28].

The top 12% nodes with the highest degree in the HC group based on the individual susceptibility network were defined as hub nodes [29–31]. Based on this categorization, the whole brain regions were classified into hub and peripheral nodes. The connections of each individual’s susceptibility network were divided into three types: “rich-club connections”, which linked hub nodes to other hub nodes; “feeder connections”, which linked hub nodes to peripheral nodes; and “local connections”, which linked peripheral nodes to other peripheral nodes [26, 32].

#### Feature construction and pattern classification

To conduct both feature selection and classification, we employ sparse logistic regression (SLR) based on the ${l}_{1}$-norm regularization (https://www.public.asu.edu/~jye02/Software/SLEP). Let ${{\bf{x}}}_{i}\in {{\mathbb{R}}}^{d}$ be the input feature with *d* dimension, ${y}_{i}\in \left\{-\mathrm{1,1}\right\}$ be the binary label of the $i$-th sample and ${\bf{W}}{{\in }}{{\mathbb{R}}}^{d}$ be the weighted coefficient. The sparse logistic regression is defined as follows:

3 $$\frac{1}{n}\mathop{\sum }\limits_{i}^{n}\log \left(1+\exp \left(-{y}_{i}\left({{\bf{W}}}^{T}{{\bf{x}}}_{i}+{\bf{b}}\right)\right)\,\right)+\lambda {{\bf{||W||}}}_{1}$$

where the sparse level of the ${\bf{W}}$ is controlled by the hyperparameter $\lambda$. **b** is the intercept, ${{\boldsymbol{||}}{{\cdot }}{\boldsymbol{||}}}_{{\boldsymbol{1}}}$ is the ${l}_{1}$-norm and n is the number of participants. In particular, a leave-one-out cross-validation strategy is conducted to validate its result, where the hyperparameter is empirically setting to 0.01. The resulting optimization problem is convex and nonsmooth. We also reported the selection feature, in which the parameter ${W}_{j}\,\ne\, 0$. The Accuracy, sensitivity, specificity, and AUC of the receiver operating characteristic curve (ROC) were applied to evaluate the classification performance.

### Statistical analysis

Demographic and neuroimaging characteristics and cognitive assessments were compared among the HC, MCI-AD, WMH-NC, and WMH-MCI groups using chi-square tests, Mann‒Whitney U tests and one-way analysis of variance (ANOVA) when appropriate using the Statistical Package for Social Sciences (SPSS V22).

The group differences in the global and nodal metrics of the susceptibility network between the HC and MCI-AD groups were assessed by two-sample *t* tests, while those among the HC, WMH-NC, and WMH-MCI groups were examined by ANOVA after controlling for potential confounders (including the effect of age, gender and years of education). The statistically significant differences in the regional properties and edges were determined using the nonparametric permutation test (5000 permutation times) based on FSL’s Randomise program. To characterize the distribution patterns of these altered susceptibility connectivity, brain regions from the BNA were mapped into the subcortical network (SN) and another seven functional networks proposed by Yeo et al. [33] based on an in-house Matlab script. Indeed, each voxel in the BNA corresponds to a network label of Yeo-7 networks. The Yeo-7 networks atlas do not include the SN, so the subcortical areas of new atlas are as same as it from the BNA. The eight intrinsic brain networks included the visual network (VN), somatomotor network (SMN), dorsal attention network (DAN), salience/ventral attention network (SVAN), limbic network (LN), frontoparietal network (FPN), default mode network (DMN) and SN.

Mediation analyses were used to assess whether altered susceptibility connections mediated the relationships between WMH and cognition after controlling for the effects of age, gender, and years of education. We computed bias-corrected 95% confidence intervals (CI) for the size of the mediating effects with bootstrapping (k = 5000 samples). The mediation analyses were conducted by using the PROCESS package (http://www.afhayes.com/) in SPSS software.

Furthermore, functional and genetic annotation analyses were also preliminarily performed to provide a biological interpretation of the altered susceptibility connectivity by using the Brain Annotation Toolbox (https://github.com/zhaowenliu/BAT). For a subnetwork, the extent of activation for a given functional term from the Neurosynth database (https://www.neurosynth.org/) was defined as the mean coactivation ratio. Gene expression data were acquired from the Allen Human Brain Atlas (AHBA, https://human.brain-map.org/), which provides normalized microarray gene expression data for six postmortem adult brains. Finally, enrichment analyses for genes consistently associated with susceptibility connectivity alterations were performed with ToppGene software (https://toppgene.cchmc.org/).

### Validations: reproducibility and test-retest reliability

#### Brain parcellation effects

Two widely used brain templates (i.e., the AAL and BNA) defined network nodes, which could validate our findings in different brain parcellation templates.

#### Network type effects

The similarity matrices were thresholded into both binary and weighted networks to reduce the effects on topological properties.

#### Test–retest reliability

We explored the test-retest (TRT) reliability of this method in constructing the susceptibility network based on different cortical parcellation approaches from the group level and individual level. The test-retest dataset consisted of 15 participants (mean age: 64.38 ± 8.35 years; HC = 7, MCI-AD = 2, WMH-NC = 5, WMH-MCI = 1) who were each scanned twice with an average interval of 11 months. In addition, their cognitive performance remained unchanged, and no neuropsychiatric disease occurred between the two MRI sessions. At the group level, quantitative spatial correlation analyses were used to assess the similarity of mean susceptibility network matrices (i.e., KLSE matrices) between the first session and the second session. Specifically, for each cortical parcellation approach, a scatter diagram and pearson correlation coefficient showed the correlation between the two 90 × 90 or 246 × 246 matrices. At the individual level, the reliability of each connectivity element was estimated using the intraclass correlation coefficient (ICC, two-way mixed single measures testing for consistency) for each cortical parcellation scheme as defined as follows [34]:

4 $${\rm{ICC}}=\frac{{{MS}}_{B}-{{MS}}_{E}}{{{MS}}_{B}+\left(k-1\right){{MS}}_{E}}$$

where *k* is the number of repeated sessions (i.e., *k* = 2), *MS*_*B*_ is the between-subjects mean square and *MS*_*E*_ is the error mean square. The ICC is close to 1 if the measurements of two sessions are consistent for each subject in the sample and 0 otherwise. The ICC was calculated based on a MATLAB function (https://ww2.mathworks.cn/matlabcentral/fileexchange/22099-intraclass-correlation-coefficient-icc).

## Results

### Demographic and clinical characteristics

The demographic and clinical data of the HC and those in the MCI-AD, WMH-NC, and WMH-MCI groups are shown in Table 1. One-way ANOVA or the χ^2^ test indicated that the four groups were not matched for age (*F* = 12.699, *p* < 0.001), years of education (*F* = 3.782, *p* = 0.011), and gender distribution (*χ*^*2*^ = 14.150, *p* = 0.003). It should be noted that the effects of age, gender and years of education were controlled for in the following network analyses. There were no significant differences in the GMV/TIV ratio among the four groups (*F* = 1.403, *p* = 0.243) or WMH volume between the WMH-NC and WMH-MCI groups (*Z* = −0.010, *p* = 0.990). In addition, MMSE (*F* = 22.226, *p* < 0.001), MoCA-BJ (*F* = 46.485, *p* < 0.001), episodic memory (*F* = 25.539, *p* < 0.001), visuospatial processing function (*F* = 21.111, *p* < 0.001), information processing speed (*F* = 18.319, *p* < 0.001), language function (*F* = 21.690, *p* < 0.001) and executive function (*F* = 26.410, *p* < 0.001) were significantly lower in the MCI-AD group and WMH-MCI group.

**Table 1:** Demographic and neuropsychological data.

Items	HC (*n* = 76)	MCI-AD (*n* = 58)	WMH		*F/χ2/Z*	*p* value
			NC (*n* = 31)	MCI (*n* = 35)		
Demographics						
Age (years)	61.882 ± 7.811	62.500 ± 8.925	69.258 ± 7.421	70.029 ± 8.049	12.699	<0.001^a,b^
Education (years)	12.013 ± 4.675	10.414 ± 2.702	9.419 ± 5.835	9.357 ± 4.059	3.782	0.011^a,^^b^
Gender (male/female)	47/29	22/36	12/19	10/25	14.150	0.003^c,^^b^
Neuroimaging characteristics						
GMV/TIV ratio	0.352 ± 0.036	0.361 ± 0.037	0.353 ± 0.032	0.346 ± 0.040	1.403	0.243^a^
WMH (ml)	–	–	11.240 (6.080, 17.630)	10.750 (6.220, 20.130)	−0.010	0.990^d^
General cognition						
MMSE	28.513 ± 1.400	27.603 ± 1.643	27.484 ± 2.515	24.457 ± 4.488	22.226	<0.001^a,^^b^
MoCA-BJ	25.895 ± 2.595	21.293 ± 2.791	24.613 ± 3.905	18.829 ± 4.508	46.485	<0.001^a,^^b^
Composition Z scores of each cognitive domain						
Episodic memory	0.458 ± 0.763	−0.388 ± 0.693	0.308 ± 0.753	−0.624 ± 0.716	25.539	<0.001^a,^^b^
AVLT-DR	5.237 ± 2.274	3.483 ± 1.740	4.903 ± 2.022	3.029 ± 1.618	14.666	<0.001^a,^^b^
VR-DR (WMS)	9.303 ± 2.643	6.293 ± 3.335	8.806 ± 3.301	5.400 ± 2.851	19.768	<0.001^a,^^b^
Visuospatial function	0.332 ± 0.474	−0.051 ± 0.809	0.242 ± 0.451	−0.851 ± 1.215	21.111	<0.001^a,^^b^
CDT	3.763 ± 0.513	3.362 ± 0.810	3.677 ± 0.541	2.686 ± 1.078	18.553	<0.001^a,^^b^
VR-C	13.763 ± 0.671	13.328 ± 1.751	13.645 ± 0.608	12.114 ± 2.386	10.922	<0.001^a,^^b^
Information processing speed	0.435 ± 0.775	−0.182 ± 0.696	−0.002 ± 0.762	−0.641 ± 0.754	18.319	<0.001^a,^^b^
TMT-A (inverse)	0.021 ± 0.009	0.017 ± 0.006	0.017 ± 0.007	0.014 ± 0.007	8.198	<0.001^a,^^b^
Stroop A (inverse)	0.060 ± 0.015	0.049 ± 0.015	0.054 ± 0.015	0.041 ± 0.016	13.506	<0.001^a,^^b^
Stroop B (inverse)	0.051 ± 0.014	0.040 ± 0.014	0.044 ± 0.014	0.034 ± 0.014	15.278	<0.001^a,^^b^
Language function	0.498 ± 0.712	−0.347 ± 0.753	0.067 ± 0.727	−0.566 ± 0.871	21.690	<0.001^a,^^b^
CVF	19.079 ± 4.931	14.052 ± 4.415	16.613 ± 3.694	14.257 ± 4.395	16.779	<0.001^a,^^b^
BNT	52.987 ± 5.587	47.966 ± 6.308	50.290 ± 7.313	44.486 ± 8.340	14.904	<0.001^a,^^b^
Executive function	0.434 ± 0.671	−0.268 ± 0.520	0.140 ± 0.764	−0.623 ± 0.643	26.410	<0.001^a,^^b^
DST-backward	5.079 ± 1.283	4.069 ± 0.934	4.677 ± 1.107	3.314 ± 1.301	20.752	<0.001^a,^^b^
TMT-B (inverse)	0.012 ± 0.005	0.009 ± 0.004	0.010 ± 0.005	0.007 ± 0.003	10.504	<0.001^a,^^b^
Stroop C (inverse)	0.036 ± 0.011	0.027 ± 0.009	0.033 ± 0.012	0.026 ± 0.013	9.890	<0.001^a,^^b^

Values are presented as the mean ± standard deviation (SD) or median (interquartile ranges).

^a^The *p* value was obtained by one-way ANOVA.

^b^Indicates a statistical difference between groups, *p* < 0.05.

^c^The *p* value was obtained by *χ*^2^ test.

^d^The *p* value was obtained by Mann–Whitney *U* test.

*HC* health control, *NC* normal cognition, *MCI-AD* mild cognitive impairment due to Alzheimer’s disease, *GMV* gray matter volume, *TIV* total intracranial volume, *WMH* white matter hyperintensities, *MMSE* mini mental state examination, *MoCA-BJ* Beijing version of the Montreal Cognitive Assessment, *AVLT-DR* Auditory Verbal Learning Test-delayed recall, *VR-DR* visual reproduction-delay recall, *WMS* Wechsler Memory Scale, *CDT* Clock Drawing Test, *VR-C* visual reproduction-copy, *CVF* category verbal fluency, *BNT* Boston Naming Test, *DST* Digit Span Test, *TMT-A and TMT-B* Trail Making Test-A and B, *Stroop A, B and C* Stroop Color and Word Tests A, B, and C.

### The TRT reliability of KLSE-based susceptibility connectivity matrices

**Fig. 2:**
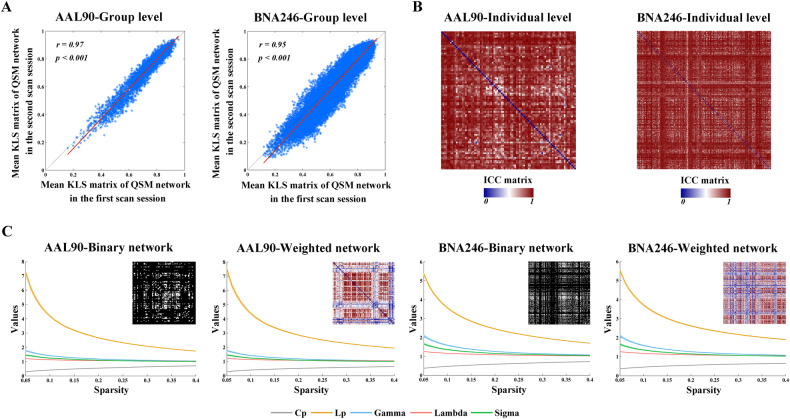
TRT reliability and small-worldness of KLSE-based susceptibility network. **A** The overall patterns of mean susceptibility network matrices were highly similar between the first session and the second session regardless of the different parcellation schemes. **B** Good reliability for interregional KLSE values in the different parcellation schemes. **C** The susceptibility network in the HC group fulfilled Gamma (*γ*) > 1, Lambda (*λ*) ≈ 1, and Sigma (*σ*) > 1 under each analytical combination of network type and brain parcellation scheme. AAL Anatomical Automatic Labeling, BNA Brainnetome atlas, QSM quantitative susceptibility mapping, KLS Kullback-Leibler divergence similarity, ICC intra-class correlation coefficient, *C*_*p*_ clustering coefficient, *L*_*p*_ characteristic path length.

The test-retest dataset consisted of 15 subjects (mean age: 64.00 ± 8.50 years; HC = 7, MCI = 2, WMH-NC = 5, WMH-MCI = 1) who were each scanned twice with an average interval of 11 months. At the group level, the quantitative spatial correlation analysis showed that the mean susceptibility network matrices were highly similar between the first scan and the second scan regardless of the different parcellation schemes (AAL: *r* = 0.97, *p* < 0.001; BNA: *r* = 0.94, *p* < 0.001; Fig. 2A). At the individual level, ICC-based TRT reliability analysis of the susceptibility network matrices indicated good reliability for interregional KLSE values (AAL: 0.70 ± 0.20; BNA: 0.62 ± 0.22; Fig. 2B).

### Small-world property of the susceptibility network

As shown in Fig. 2C, over the threshold range of 0.05 to 0.4, the susceptibility network in the HC group fulfilled Gamma (*γ*) > 1, Lambda (*λ*) ≈ 1, and Sigma (*σ*) > 1 independent of the network type and brain parcellation template. These findings demonstrated the small-world property of the brain susceptibility network.

### Altered global properties of the susceptibility network

**Fig. 3:**
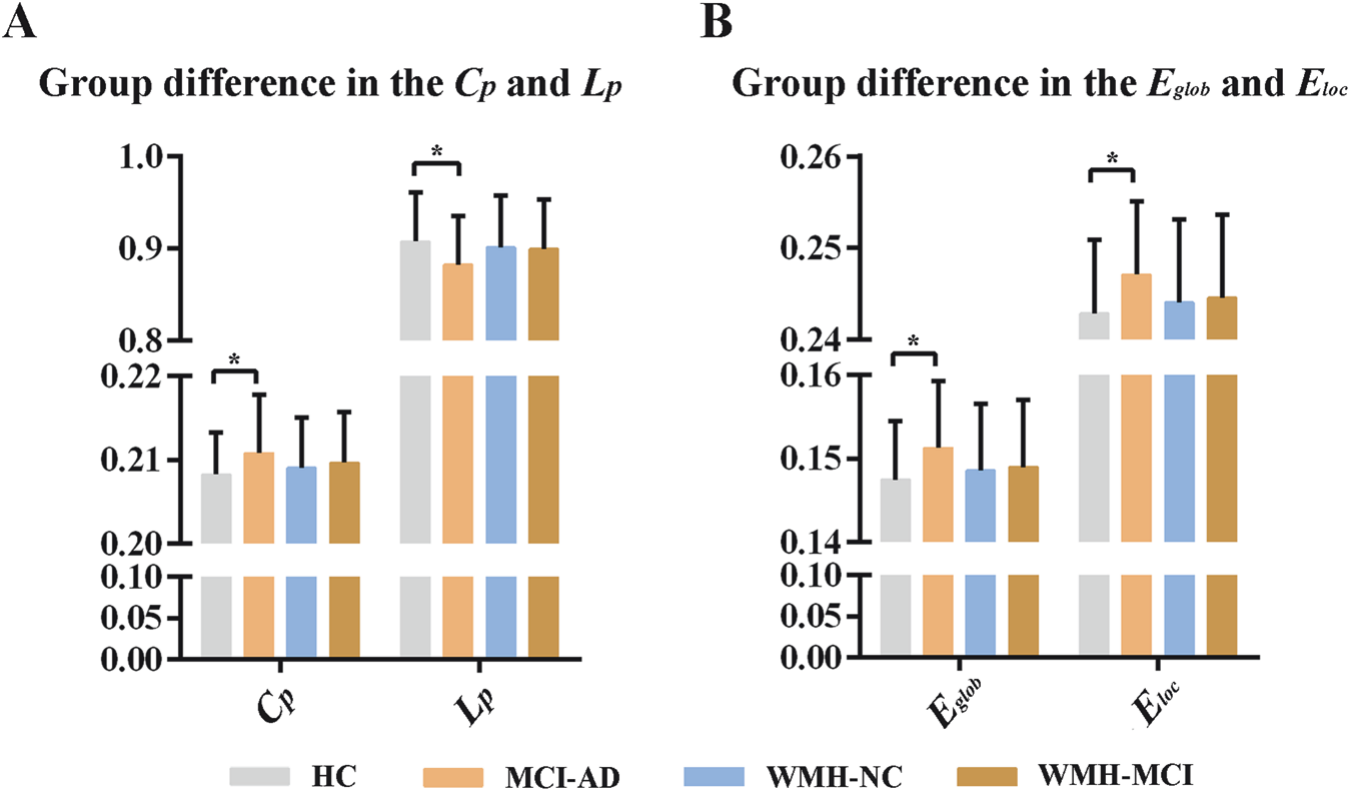
Global properties of the susceptibility network. **A** The MCI-AD group showed significantly increased clustering coefficient (*t* = 5.878, *p* = 0.017) and decreased characteristic path length (*t* = 7.081, *p* = 0.009) compared to the HC group. **B** The MCI-AD group showed significantly increased global efficiency (*t* = 6.463, *p* = 0.012) and local efficiency (*t* = 1.403, *p* = 0.010) relative to the HC group. * indicates a statistical difference between groups, *p* < 0.05. Cp clustering coefficient, Lp characteristic path length, Eglob global efficiency, Eloc local efficiency, HC health control, NC normal cognition, MCI-AD mild cognitive impairment due to Alzheimer’s disease, WMH white matter hyperintensities.

The significant differences in small-world properties and network efficiency between the MCI-AD group and the HC group are shown in Fig. 3. The MCI-AD group showed a significantly increased clustering coefficient (*t* = 5.878, *p* = 0.017; Fig. 3A), global efficiency (*t* = 6.463, *p* = 0.012; Fig. 3B) and local efficiency (*t* = 1.403, *p* = 0.010; Fig. 3B) relative to the HC group. The characteristic path length was shorter in the MCI-AD group than in the HC group (*t* = 7.081, *p* = 0.009; Fig. 3A). However, no significant difference in global properties was found between patients with WMH and controls (Fig. 3). These results were obtained under the binary networks constructed by the BNA template. The results obtained based on the AAL template and the weighted networks were similar to these findings (Supplementary Materials).

### Altered regional properties of the susceptibility network

**Fig. 4:**
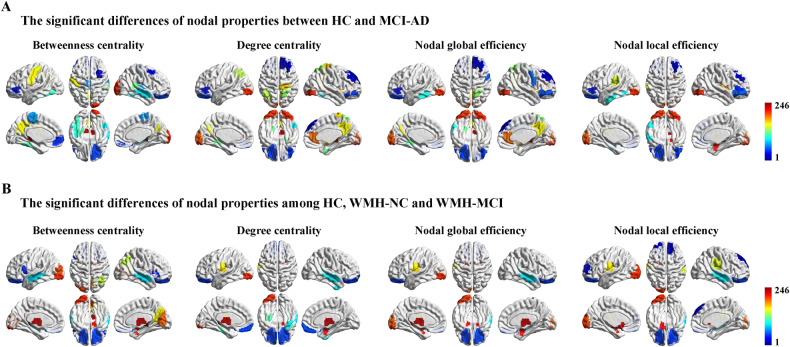
Regional properties of the susceptibility network. **A** The MCI-AD group showed significantly altered nodal properties predominantly located in the DMN and VN, such as superior frontal gyrus, precuneus, middle temporal gyrus and occipital gyrus. **B** Alterations of regional properties in SN, LN, and VN were identified in patients with WMH. HC health control, NC normal cognition, MCI-AD mild cognitive impairment due to Alzheimer’s disease, WMH white matter hyperintensities, DMN default mode network, VN visual network, SN subcortical network, LN limbic network. The abbreviations of 246 brain regions could be seen in Supplemental Table 2.

Nonparametric permutation tests (*p* < 0.05, uncorrected) were used to preliminarily reveal the abnormal regional metrics of the susceptibility network in the MCI-AD and WMH-MCI groups (Fig. 4). The MCI-AD group showed significantly altered nodal properties predominantly located in the DMN and VN, such as the superior frontal gyrus, precuneus, middle temporal gyrus and occipital gyrus (Fig. 4A). In contrast, alterations in regional properties in the SN, LN, and VN were identified in patients with WMH (Fig. 4B). However, these results did not survive multiple comparison correction (e.g., Bonferroni or false discovery rate correction [FDR]). Interestingly, there was a significant trend toward exhibiting different patterns of regional properties between MCI-AD and WMH individuals. These results were obtained under the binary networks constructed by the BNA template. The results obtained based on the AAL template and the weighted networks are also shown in the Supplementary Materials.

### Rich-club organization analysis

**Fig. 5:**
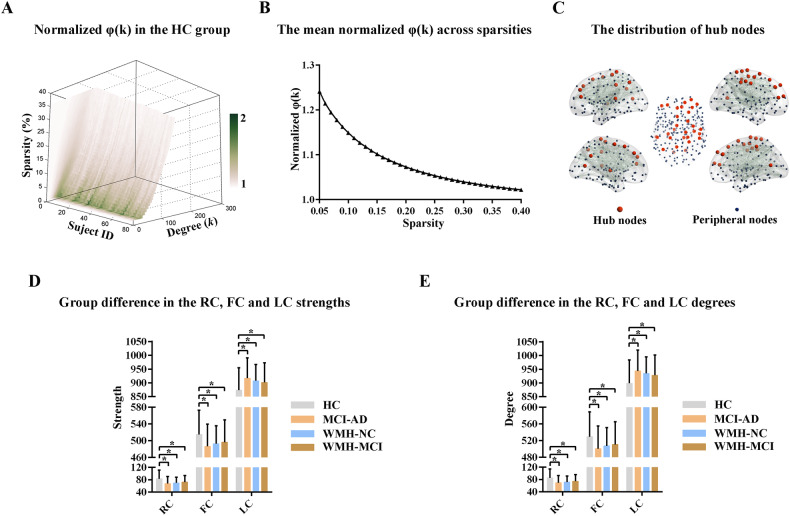
Rich-club organization analysis. **A**, **B** Rich-club organization was evident in the susceptibility network of human brain, with the normalized *φ*(*k*) > 1 over a range of sparsity from 0.05 to 0.4. **C** The distribution of hub regions. **D**, **E** Significant differences in the strength and degree of the rich-club, feeder and local connections were identified in the MCI-AD and WMH group. * indicates a statistical difference between groups, *p* < 0.05. HC health control, NC normal cognition, MCI-AD mild cognitive impairment due to Alzheimer’s disease, WMH white matter hyperintensities, RC rich-club connections, FC feeder connections, LC local connections.

As shown in Fig. 5A, B, rich-club organization was evident in the susceptibility network of the human brain, with normalized *φ*(*k*) > 1 over a range of sparsity from 0.05 to 0.4. These hub regions were primarily located in the bilateral superior frontal gyrus, middle frontal gyrus, inferior parietal lobule, precuneus, and precentral and postcentral gyrus, which were similar to previous research (Fig. 5C) [35]. In the MCI-AD group, the strength and degree of the rich-club and feeder connections were significantly lower (strength: rich-club *p* < 0.001, feeder *p* = 0.002; degree: rich-club *p* < 0.001, feeder *p* = 0.002), while the strength and degree of the local connections were significantly greater than those in the HC group (strength: local *p* = 0.001; degree: local *p* = 0.001) (Fig. 5D, E). In addition, individuals with WMH exhibited a pattern of rich-club organization similar to that of the MCI-AD group. These results were obtained under the binary networks constructed by the BNA template. The detailed data are described in Supplementary Table 4. The results obtained based on the AAL template and the weighted networks are also shown in the Supplementary Materials.

### The distinct pattern of susceptibility connectivity in individuals with cognitive impairment

**Fig. 6:**
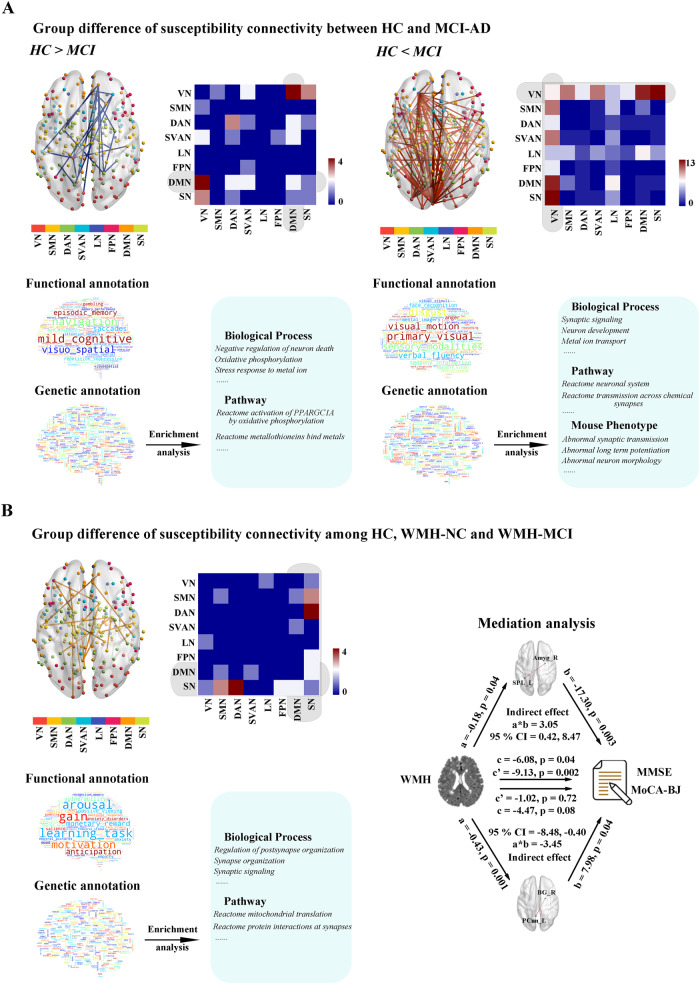
The distinct pattern of susceptibility connectivity in individuals with cognitive impairment. **A** The MCI-AD group showed decreased susceptibility edges (*p* < 0.001, permutation test), primarily involved in the DMN-related connectivity. The increased susceptibility edges were found in the MCI-AD group compared with the HC group (*p* < 0.001, *p*ermutation test), mainly distributed between VN and other networks. Results of functional and genetic annotations were as shown in main manuscript. **B** The altered susceptibility connectivity in individuals with WMH was shown by more connectivity involved in SN- and DMN-related connectivity (*p* < 0.001, permutation test). Results of functional and genetic annotations were as shown in main manuscript. The relationship between WMH and MMSE and MoCA-BJ was mediated by the susceptibility connectivity. HC health control, NC nomal cognition, MCI-AD mild cognitive impairment due to Alzheimer’s disease, WMH white matter hyperintensities, VN visual network, SMN somatomotor network, DAN dorsal attention network, SVAN salience/ventral attention network, LN limbic network, FPN frontoparietal network, DMN default mode network, SN subcortical network, MMSE Mini-Mental State Examination, MoCA-BJ Beijing version of the Montreal Cognitive Assessment, CI confidence intervals, SPL_L left superior parietal lobule, Amyg_R right amygdala, PCun_L left precuneus, BG_R right basal ganglia.

Figure 6A displays the altered susceptibility connectivity in the MCI-AD group. The MCI-AD group showed 24 decreased susceptibility edges (*p* < 0.001, permutation test), which were primarily involved in DMN-related connectivity. This dysregulated network of 24 edges was significantly enriched in 2 functional terms, including ‘mild cognitive’ (*p* = 0.014, permutation test) and ‘navigation’ (*p* = 0.042, permutation test). For the genetic analysis, we selected those connections with associated brain regions that had more than 5 AHBA samples and whose remaining 7 of the 24 edges were used for genetic analysis. In total, 4944 genes were significantly coexpressed (*p* < 0.05, FDR corrected) in the regions connected by these 7 edges. These genes were significantly related to biological processes such as ‘negative regulation of neuron death’ (*p* = 4.26e^−3^, FDR corrected), ‘oxidative phosphorylation’ (*p* = 9.16e^−3^, FDR corrected) and ‘stress response to metal ion’ (*p* = 4.79e^−3^, FDR corrected). The gene pathway ‘reactome activation of PPARGC1A by oxidative phosphorylation’ (*p* = 3.27e^−2^, FDR corrected) and ‘reactome metallothioneins bind metals’ (*p* = 4.86e^−2^, FDR corrected) were also found to be significantly enriched.

In contrast, 107 increased susceptibility edges were detected in the MCI-AD group relative to the HC group (*p* < 0.001, permutation test; Fig. 6A). These connections were mainly distributed between the VN and other networks. The term ‘primary visual’ (*p* = 0.006, permutation test) was found to be significantly associated with these altered susceptibility edges. Sixty of the 107 edges for genetic analysis and 11568 genes were found to be significantly overexpressed (*p* < 0.05, FDR corrected). These genes were significantly related to biological processes such as ‘synaptic signaling’ (*p* = 1.20e^−28^, FDR corrected), ‘neuron development’ (*p* = 1.20e^−28^, FDR corrected) and ‘metal ion transport’ (*p* = 2.48e^−17^, FDR corrected). The gene pathway ‘reactome neuronal system’ (*p* = 1.58e^−9^, FDR corrected) and ‘reactome transmission across chemical synapses’ (*p* = 1.40e^−7^, FDR corrected) were also found to be significantly enriched. These genes are also associated with abnormal mouse phenotypes, such as ‘abnormal synaptic transmission’ (*p* = 4.49e^−7^, FDR corrected), ‘abnormal long term potentiation’ (*p* = 1.21e^−7^, FDR corrected) and ‘abnormal neuron morphology’ (*p* = 1.03e^−6^, FDR corrected).

Interestingly, the altered susceptibility connectivity (17 edges) in individuals with WMH was shown by increased connectivity involved in SN- and DMN-related connectivity (*p* < 0.001, permutation test; Fig. 6B). This dysregulated network of 17 edges was significantly enriched in 48 functional terms, including ‘learning task’ (*p* < 0.001, permutation test) and ‘gain’ (*p* < 0.001, permutation test). In total, 3773 genes were identified to be significantly coexpressed (*p* < 0.05, FDR corrected) in the regions connected by 3 of the 17 edges. These genes were significantly related to biological processes such as ‘regulation of postsynapse organization’ (*p* = 4.47e^−3^, FDR corrected) and ‘synapse organization’ (*p* = 6.01e^−3^, FDR corrected). The gene pathway ‘reactome mitochondrial translation’ (*p* = 3.31e^−3^, FDR corrected) and ‘reactome protein interactions at synapses’ (*p* = 9.41e^−3^, FDR corrected) were also found to be significantly enriched.

In addition, the relationship between WMH volume and MMSE score was mediated by susceptibility connectivity between the left superior parietal lobule and right amygdala (indirect effect: 3.05; 95% CI: 0.42, 8.47; Fig. 6B). The indirect effect of WMH volume on MoCA-BJ score was significantly mediated by susceptibility connectivity between the left precuneus and right basal ganglia (indirect effect: −3.45; 95% CI: −8.48, −0.40; Fig. 6B).

### Discriminative analysis

**Fig. 7:**
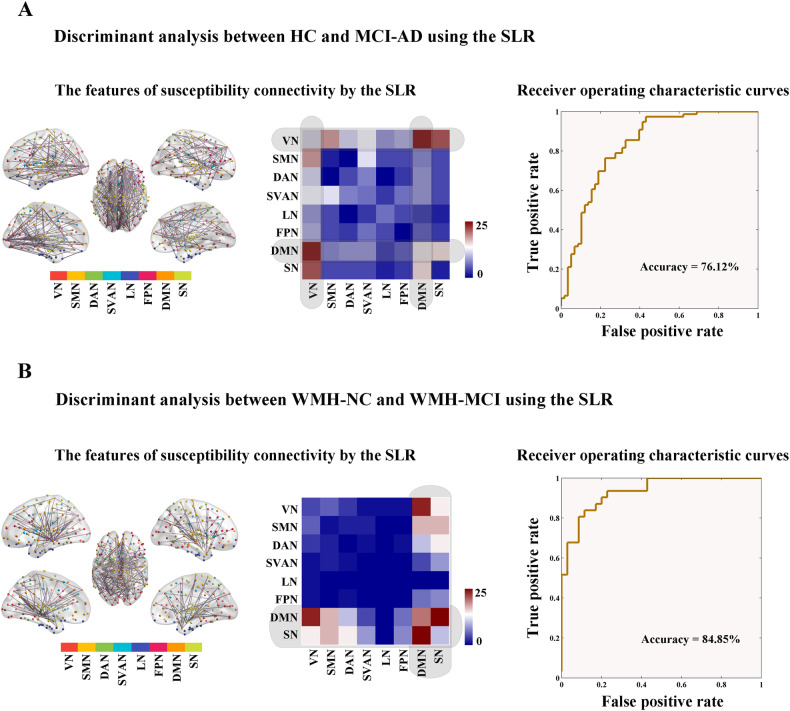
Susceptibility connectivity as features for identifying individuals with cognitive impairment. **A** For the HC vs MCI-AD classification, 263 edges as the features selected by the SLR are primarily distributed throughout the DMN-related and VN-related connectivity. The cross validation accuracy and AUC were 76.12% and 0.83, with a sensitivity and specificity of 76.32% and 75.86%, respectively. **B** For the WMH-NC vs WMH-MCI classification, 199 edges as the features selected by the SLR are primarily distributed throughout the SN-related and DMN-related connectivity. The cross validation accuracy and AUC were 84.85% and 0.93, with a sensitivity and specificity of 87.10% and 82.86%, respectively. HC health control, NC normal cognition, MCI-AD mild cognitive impairment due to Alzheimer’s disease, WMH white matter hyperintensities, VN visual network, SMN somatomotor network, DAN dorsal attention network, SVAN salience/ventral attention network, LN limbic network, FPN frontoparietal network, DMN default mode network, SN subcortical network, SLR sparse logistic regression, AUC area under curve.

In this study, an SLR based on ${l}_{1}$-norm regularization was used to select features of susceptibility connectivity for identifying individuals with cognitive impairment. For the HC vs. MCI-AD classification, 263 edges, as the features selected by the SLR, were primarily distributed throughout the DMN-related and VN-related connectivity (Fig. 7A). The cross-validation accuracy and AUC were 76.12% and 0.83, respectively (sensitivity = 76.32%, specificity = 75.86%; Fig. 7A). For the WMH-NC vs. WMH-MCI classification, 199 edges as features were involved in SN-related and DMN-related connectivity (Fig. 7B). The cross-validation accuracy and AUC were 84.85% and 0.93, respectively (sensitivity = 87.10%, specificity = 82.86%; Fig. 7B).

## Discussion

Our study proposed an individual-level susceptibility network construction approach based on QSM imaging and systematically revealed the topological organization of the two most common aetiologies of cognitive impairment (i.e., AD and VCI). We proposed that the brain susceptibility network was naturally organized in a small-world manner with highly connected hubs. More importantly, characteristic patterns of abnormal susceptibility networks were found in both MCI-AD and WMH-MCI and could be applied to differentiate between individuals with cognitive impairment and healthy elderly individuals.

Previous studies based on QSM data have concentrated on local susceptibility alterations via region-based methods in neuroscience [8, 36]. However, the interregional relationships in brain susceptibility have never been investigated. In AD, neuroimaging studies using PET showed a spatial correspondence between the spatial distribution of Aβ and the topography of the DMN and FPN [37–39]. These findings supported the ‘prion-like’ spreading hypothesis that pathogenic proteins originate as prion-like seeded aggregations, which then spread along the neuronal pathways that compose macroscopic brain networks [40]. Previous studies have reported the positive correlations between iron load and Aβ accumulation mainly located in the frontotemporal cortex in AD patients and healthy old-aged adults [2, 8, 41]. Therefore, iron deposition may be closely related to Aβ load and Aβ load could be transported to separate brain regions through axons. We infer that closely connected brain regions exhibit similar patterns of iron deposition. In this work, we introduced a novel framework to construct a susceptibility network at the individual level based on the KLSE model, which estimates the similarity of susceptibility between any two brain regions. This approach not only considered the variability and complexity of the brain cortex structure but also allowed us to quantify the susceptibility relationships in each individual [11]. We speculate that the relatively high similarity of iron distributions in any two brain regions reflects relatively more interregional information transmission. Based on datasets that were scanned twice, we investigated the TRT reliability of the KLSE method for constructing susceptibility networks through different cortical parcellation approaches. Our results showed that the interregional KLSE values exhibited good TRT reliability independent of brain parcellation and network type. Therefore, we speculated that KLSE-based brain susceptibility network analysis could be a plausible approach for future investigations of the neuroimaging phenotypes of cognitive impairment.

Generally, the small-world attribute of brain networks offers a structural substrate for functional integration and segregation to facilitate rapid information communication throughout the whole brain [25]. Consistent with the findings of other neuroimaging modalities, the susceptibility network constructed by QSM imaging globally showed high efficiency, small-worldness, and rich-club organization in the present study [42–44]. Furthermore, the hub regions identified in our study were primarily located in DMN-related regions, which was largely similar to the findings of previous research [35]. Our findings suggest that the susceptibility network is a complex but efficient neuronal architecture that reflects an optimal balance between functional integration and segregation. In accordance with this observation, KLSE-based susceptibility network analysis could be considered a potential approach to investigating topological organization in cognitive neuroscience and neuropsychology.

The distinct features of the whole-brain susceptibility network were explored and compared between the MCI-AD and WMH-MCI groups. The MCI-AD group exhibited a significantly greater clustering coefficient and shorter characteristic path length than did the controls. The global properties exhibited similar but not significantly different trends in the WMH-MCI group. Consistent with previous findings in Aβ brain networks, the increased segregation and preserved integration of the susceptibility network may reveal a regularization pattern of altered small-worldness in MCI-AD patients [45]. Duan et al. [45] demonstrated that the Aβ brain network constructed from PET images exhibited more noticeable small-world attributes in AD patients than in HC, which could be attributed to the Aβ cascade hypothesis. As proposed in previous research, elevated brain iron deposition occurs in AD patients and is positively related to Aβ burden [2, 41]. Due to the susceptibility values reflecting the distribution of brain iron, the altered global properties of the susceptibility network showed a similar pattern to those of the Aβ brain network.

In addition, increased local connections were found, whereas decreased rich-club and feeder connections were detected in the MCI-AD and WMH-MCI patients. Cascading network dysfunction was proposed for AD: deterioration begins with local overload and then transfers to other regions, including hub nodes, eventually resulting in widespread impairment [46]. Evidence from the brain structural network suggested that connectivity among peripheral nodes was predominantly disrupted, but rich-club connectivity remained relatively preserved in the early stage of AD [47]. Similar findings were presented in individuals with WMH-MCI, which revealed apparent disruptions in peripheral morphological connectivity [48]. More importantly, this phenomenon was also observed in structural connectivity constructed by DTI in AD patients with WMH [49]. As a result, the MCI-AD and WMH-MCI patients exhibited similar patterns of rich-club properties in the susceptibility network from the perspective of rich-club architecture.

Depicting connectivity patterns in the human brain has become an important topic in neuroscience. Common and specific patterns of abnormal susceptibility connectivity were found in both MCI-AD and WMH-MCI patients. The altered susceptibility connectivity in the MCI-AD patients indicated relatively more connectivity involved in VN-related and DMN-related connectivity, while more altered SN-related and DMN-related connectivity was found in the WMH-MCI patients. The functional annotation analyses indicated that these altered susceptibility connectivity was significantly enriched in cognition-related terms. These characteristic connectivity might play an important role in cognitive processing, which were consistent with previous studies from different neuroimaging modals. Differential diagnosis between AD and VCI is quite difficult because their pathophysiological mechanisms overlap as well as their concurrence. Notably, these characteristic patterns of abnormal susceptibility connectivity could distinguish MCI-AD and WMH-MCI patients from HC based on the SLR. Voxel-based QSM analyses indicated increased magnetic susceptibility values mainly in the superior temporal gyrus, middle frontal gyrus, parahippocampal gyrus, posterior cingulate, precentral gyrus, and caudate body, which largely overlapped with the DMN in AD patients compared to HC [21]. Additionally, QSM better differentiated amnestic MCI from HC than gray matter volume in the regions where iron and Aβ accumulate in the posterior cingulate cortex, entorhinal cortex, precuneus and neocortex [21]. Rao et al. [50] reviewed the relationship between the presence of brain iron burden and glucose hypometabolism in different brain regions in AD. They found that a combined pattern of brain iron burden and glucose hypometabolism predominantly occurred in specific affected regions (e.g., temporal cortex, hippocampus and parietal cortex), which largely overlapped with the DMN [50]. These findings from regional QSM analysis provided potential evidence to support the hypothesis that altered DMN-related susceptibility connectivity plays a crucial role in AD pathogenesis. However, no VN-related QSM abnormalities have been reported in previous studies via regional susceptibility measures. The gene annotation analysis revealed that those genes related to altered susceptibility connectivity were enriched mainly for synaptic function, consistent with previous findings in AD [51]. We speculated that the susceptibility network could supplement additional information referring to the interregional associations that are not evident from regional QSM analysis.

WMH, as the main imaging manifestation of small vessel disease, is thought to be the primary cause of VCI. Complex cognitive processing depends on information transmission through multiple brain networks. Convergent evidence has indicated that altered brain networks, especially the FPN, DMN, and SN, are closely associated with cognitive impairment in individuals with WMH [48, 52–54]. Combining voxel-based lesion-symptom mapping with ROI-based Bayesian network analyses, a cohort study of elderly individuals suggested that damage to frontal-subcortical projection fibers dependent on WMH load was associated with impaired processing speed performance [52]. Compared with WMH-NC, WMH-MCI showed significantly decreased functional connectivity between subcortical nuclei and cortical hub regions of cognitive networks. Furthermore, these changes in functional connectivity could distinguish WMH-MCI from WMH-NC well based on a support vector machine classifier [53]. From the perspective of the morphological connectome, the altered topological organization related to WMH-related cognitive decline was primarily involved in the DMN and LN, which are recognized for their roles in a wide range of cognitive domains, such as memory function and executive function [48, 54]. As mentioned in the Introduction, less consistency in the presence and pattern of iron accumulation in VCI was reported, which could be due to voxel-based or region-based approaches. Consistent with and even expanding on previous results, our findings suggested that WMH-related cognitive impairment could be attributed not only to functional and structural-functional disconnections but also to altered DMN-related and SN-related susceptibility connectivity, and these characteristic patterns of susceptibility networks may provide additional evidence to enhance our understanding of the pathogenesis of WMH-related cognitive decline.

There are several limitations that still need to be further considered. First, the exact pathological and physiological meaning of the susceptibility network remains unclear. Further research is warranted to combine different imaging techniques (such as DTI, rs-fMRI, imaging genetics, animal experiments, and basic research) to explore the underlying mechanisms involved. Second, all subjects in our study lacked PET or cerebrospinal fluid examinations to identify AD pathological biomarkers such as Aβ or tau deposition, which makes it difficult to distinguish mixed dementia. Therefore, future studies are needed to supplement the pathological data to fully verify the applicability of the susceptibility connectome method. Third, the sample size was relatively small, and group differences in susceptibility connectivity did not survive multiple comparison corrections. Thus, these results should be considered exploratory. A multicentre longitudinal design including an independent cohort is needed to validate the preliminary findings.

## Conclusion

This study proposes a novel concept of the susceptibility network defined as interregional susceptibility relations and explores its potential application in cognitive impairment diseases. The shared and specific patterns of the susceptibility network identified in both MCI-AD and WMH-MCI may provide a potential diagnostic biomarker for cognitive impairment, which could enhance the understanding of the relationships between brain iron burden and cognitive decline from a network perspective.

### Supplementary information

Supplementary materials

## Data Availability

Data will be made available on request.

## Code availability

Codes will be made available on request.
