# Correction: The characteristic patterns of individual brain susceptibility networks underlie Alzheimer’s disease and white matter hyperintensity-related cognitive impairment

**DOI:** 10.1038/s41398-024-03048-x

**Published:** 2024-08-14

**Authors:** Haifeng Chen, Jingxian Xu, Weikai Li, Zheqi Hu, Zhihong Ke, Ruomeng Qin, Yun Xu

**Affiliations:** 1 Nanjing Drum Tower Hospital Clinical College of Traditional Chinese and Western Medicine, Nanjing University of Chinese Medicine, Nanjing, China; 2 Department of Neurology, Nanjing Drum Tower Hospital, Affiliated Hospital of Medical School, Nanjing University, Nanjing, China; 3 Jiangsu Key Laboratory of Molecular Medicine, Medical School of Nanjing University, Nanjing, China; 4 Jiangsu Province Stroke Center for Diagnosis and Therapy, Nanjing, China; 5 Nanjing Neuropsychiatry Clinic Medical Center, Nanjing, China; 6 School of Mathematics and Statistics, Chongqing Jiaotong University, Chongqing, China; 7 MIIT Key Laboratory of Pattern Analysis and Machine Intelligence, Nanjing, China

**Keywords:** Long-term memory, Hippocampus

Correction to: *Translational Psychiatry* 10.1038/s41398-024-02861-8, published online 04 April 2024

In this article the affiliation details for Prof. Yun Xu were incorrectly given as ‘Yun Xu 2,3,4,5’ but should have been ‘Yun Xu 1,2,3,4,5’.

The original article has been corrected.

