# Supplementary material for: The characteristic patterns of individual brain susceptibility networks underlie Alzheimer’s disease and white matter hyperintensity-related cognitive impairment
Source: Transl Psychiatry. 2024 Apr 4;14:177. doi: 10.1038/s41398-024-02861-8 (PMC10994911; doi:10.1038/s41398-024-02861-8)

**Supplementary Materials**

**Neuropsychological measurement**

All participants underwent a standardized neuropsychological test protocol, including general cognitive performance and multiple cognitive domain examinations. general cognitive performance was evaluated by Mini-Mental State Examination (MMSE) and the Beijing version of the Montreal Cognitive Assessment (MoCA-BJ). The raw test scores were converted to Z-scores, which were used to calculate the compound cognitive index. Episodic memory was calculated as the mean of the Z-scores from the Wechsler Memory Scale-Visual Reproduction-delayed recall and Auditory Verbal Learning Test-delayed recall scores. Visuospatial function is a compound score that includes the mean of the Z-scores of the Clock Drawing Test and Visual Reproduction–copy test. Information processing speed was calculated as the average Z-scores of the Trail Making Test-A and the Stroop Color and Word Tests A and B. The language function consisted of the Category Verbal Fluency test and Boston Naming Test. Executive function is a compound score of the average Z-scores of the Digit Span Test-backward, Trail Making Test-B and Stroop Color and Word Tests C. The cognitive status was determined based on the MoCA-BJ. In order to avoid the level of education affecting the results of this scale, the optimal cutoff points are determined according to education level. For subjects with no formal education, the MoCA-BJ cutoff was 13/14; for subjects with 1-6 years of education, the MoCA-BJ cutoff was 19/20; and for subjects with seven or more years of education, it was 24/25.

**MRI Scanning**

The multimodal neuroimaging data were acquired using a Philips Medical Systems 3.0T machine. The protocol included the high-resolution 3D T_1_ imaging [repetition time (TR) = 9.8 ms, echo time (TE) = 4.6 ms, ﬂip angle (FA) = 8°, field of view (FOV) = 256 × 192 mm^2^, acquisition voxel size = 1 × 1 × 1 mm^3^, number of slices = 256, thickness = 1.0 mm], a 3D fluid-attenuated inversion recovery (FLAIR) sequence [TR = 4500 ms, TE = 348 ms, FA = 90°, FOV = 190 × 259 mm^2^, acquisition voxel size = 0.95 × 0.90 × 0.90 mm^3^, number of slices = 288, thickness = 0.90 mm] and a 3D fast field-echo imaging for QSM [TR = 50 ms, first TE/ΔTE/final TE = 7.5/7.2/43.5 ms, FA = 17°, FOV = 229 × 229 mm^2^, acquisition voxel size = 0.65 × 0.65 × 2 mm^3^, number of slices = 63, thickness = 2.0 mm].

**Altered global properties of the susceptibility network in individuals with cognitive impairment**

Results based on the AAL90-Binary network, AAL90-Weighted network and BNA246-Weighted network were similar to our findings described in the main manuscript. The additional results were shown in Supplementary Figure 1.

**Altered regional properties of the susceptibility network in individuals with cognitive impairment**

Results based on the AAL90-Binary network, AAL90-Weighted network and BNA246-Weighted network were similar to our findings described in the main manuscript. The additional results were shown in Supplementary Figure 2.

**Rich-club organization analysis**

We also conducted rich-club organization analysis based on the AAL90-Binary network, AAL90-Weighted network and BNA246-Weighted network. These results were similar to our findings described in the main manuscript. The additional results were shown in Supplementary Figure 3, 4 and 5.

| **Supplemental Table 1 Brain regions and their abbreviations in AAL atlas** | | |
| --- | --- | --- |
| **Labels** | **Abbreviations** | **Brain regions** |
| 1 | PrecentralL | Precental gyrus |
| 2 | PrecentralR | Precental gyrus |
| 3 | FrontalSupL | Superior frontal gyrus, dorsolateral |
| 4 | FrontalSupR | Superior frontal gyrus, dorsolateral |
| 5 | FrontalSupOrbL | Superior frontal gyrus, orbital part |
| 6 | FrontalSupOrbR | Superior frontal gyrus, orbital part |
| 7 | FrontalMidL | Middle frontal gyrus |
| 8 | FrontalMidR | Middle frontal gyrus |
| 9 | FrontalMidOrbL | Middle frontal gyrus, orbital part |
| 10 | FrontalMidOrbR | Middle frontal gyrus, orbital part |
| 11 | FrontalInfOperL | Inferior frontal gyrus, opercular part |
| 12 | FrontalInfOperR | Inferior frontal gyrus, opercular part |
| 13 | FrontalInfTriL | Inferior frontal gyrus, triangular part |
| 14 | FrontalInfTriR | Inferior frontal gyrus, triangular part |
| 15 | FrontalInfOrbL | Inferior frontal gyrus, orbital part |
| 16 | FrontalInfOrbR | Inferior frontal gyrus, orbital part |
| 17 | RolandicOperL | Rolandic operculum |
| 18 | RolandicOperR | Rolandic operculum |
| 19 | SuppMotorAreaL | Supplementary motor area |
| 20 | SuppMotorAreaR | Supplementary motor area |
| 21 | OlfactoryL | Olfactory cortex |
| 22 | OlfactoryR | Olfactory cortex |
| 23 | FrontalSupMedialL | Superior frontal gyrus, medial |
| 24 | FrontalSupMedialR | Superior frontal gyrus, medial |
| 25 | FrontalMidOrbL | Superior frontal gyrus, medial orbital |
| 26 | FrontalMidOrbR | Superior frontal gyrus, medial orbital |
| 27 | RectusL | Gyrus rectus |
| 28 | RectusR | Gyrus rectus |
| 29 | InsulaL | Insula |
| 30 | InsulaR | Insula |
| 31 | CingulumAntL | Anterior cingulate and paracingulate gyri |
| 32 | CingulumAntR | Anterior cingulate and paracingulate gyri |
| 33 | CingulumMidL | Median cingulate and paracingulate gyri |
| 34 | CingulumMidR | Median cingulate and paracingulate gyri |
| 35 | CingulumPostL | Posterior cingulate gyrus |
| 36 | CingulumPostR | Posterior cingulate gyrus |
| 37 | HippocampusL | Hippocampus |
| 38 | HippocampusR | Hippocampus |
| 39 | ParaHippocampalL | Parahippocampal gyrus |
| 40 | ParaHippocampalR | Parahippocampal gyrus |
| 41 | AmygdalaL | Amygdala |
| 42 | AmygdalaR | Amygdala |
| 43 | CalcarineL | Calcarine fissure and surrounding cortex |
| 44 | CalcarineR | Calcarine fissure and surrounding cortex |
| 45 | CuneusL | Cuneus |
| 46 | CuneusR | Cuneus |
| 47 | LingualL | Lingual gyrus |
| 48 | LingualR | Lingual gyrus |
| 49 | OccipitalSupL | Superior occipital gyrus |
| 50 | OccipitalSupR | Superior occipital gyrus |
| 51 | OccipitalMidL | Middle occipital gyrus |
| 52 | OccipitalMidR | Middle occipital gyrus |
| 53 | OccipitalInfL | Inferior occipital gyrus |
| 54 | OccipitalInfR | Inferior occipital gyrus |
| 55 | FusiformL | Fusiform gyrus |
| 56 | FusiformR | Fusiform gyrus |
| 57 | PostcentralL | Postcentral gyrus |
| 58 | PostcentralR | Postcentral gyrus |
| 59 | ParietalSupL | Superior parietal gyrus |
| 60 | ParietalSupR | Superior parietal gyrus |
| 61 | ParietalInfL | Inferior parietal, but supramarginal and angular gyri |
| 62 | ParietalInfR | Inferior parietal, but supramarginal and angular gyri |
| 63 | SupraMarginalL | Supramarginal gyrus |
| 64 | SupraMarginalR | Supramarginal gyrus |
| 65 | AngularL | Angular gyrus |
| 66 | AngularR | Angular gyrus |
| 67 | PrecuneusL | Precuneus |
| 68 | PrecuneusR | Precuneus |
| 69 | ParacentralLobuleL | Paracentral lobule |
| 70 | ParacentralLobuleR | Paracentral lobule |
| 71 | CaudateL | Caudate nucleus |
| 72 | CaudateR | Caudate nucleus |
| 73 | PutamenL | Lenticular nucleus, putamen |
| 74 | PutamenR | Lenticular nucleus, putamen |
| 75 | PallidumL | Lenticular nucleus, pallidum |
| 76 | PallidumR | Lenticular nucleus, pallidum |
| 77 | ThalamusL | Thalamus |
| 78 | ThalamusR | Thalamus |
| 79 | HeschlL | Heschl gyrus |
| 80 | HeschlR | Heschl gyrus |
| 81 | TemporalSupL | Superior temporal gyrus |
| 82 | TemporalSupR | Superior temporal gyrus |
| 83 | TemporalPoleSupL | Temporal pole: superior temporal gyrus |
| 84 | TemporalPoleSupR | Temporal pole: superior temporal gyrus |
| 85 | TemporalMidL | Middle temporal gyrus |
| 86 | TemporalMidR | Middle temporal gyrus |
| 87 | TemporalPoleMidL | Temporal pole: middle temporal gyrus |
| 88 | TemporalPoleMidR | Temporal pole: middle temporal gyrus |
| 89 | TemporalInfL | Inferior temporal gyrus |
| 90 | TemporalInfR | Inferior temporal gyrus |
| Abbreviations: AAL, Anatomical Automatic Labeling. | | |

| **Supplemental Table 2 Brain areas and their abbreviations in Brainnetome Atlas** | | | | | |
| --- | --- | --- | --- | --- | --- |
| **Lobe** | **Gyrus** | **Left and Right Hemisphere** | **Lobe** | **Gyrus** | **Left and Right Hemisphere** |
| **Frontal Lobe** | SFG, Superior Frontal Gyrus | SFG_L(R)71 | **Parietal Lobe** | SPL, Superior Parietal Lobule | SPL_L(R)51 |
|  |  | SFG_L(R)72 |  |  | SPL_L(R)52 |
|  |  | SFG_L(R)73 |  |  | SPL_L(R)53 |
|  |  | SFG_L(R)74 |  |  | SPL_L(R)54 |
|  |  | SFG_L(R)75 |  |  | SPL_L(R)55 |
|  |  | SFG_L(R)76 |  | IPL, Inferior Parietal Lobule | IPL_L(R)61 |
|  |  | SFG_L(R)77 |  |  | IPL_L(R)62 |
|  | MFG, Middle Frontal Gyrus | MFG_L(R)71 |  |  | IPL_L(R)63 |
|  |  | MFG_L(R)72 |  |  | IPL_L(R)64 |
|  |  | MFG_L(R)73 |  |  | IPL_L(R)65 |
|  |  | MFG_L(R)74 |  |  | IPL_L(R)66 |
|  |  | MFG_L(R)75 |  | Pcun, Precuneus | PCun_L(R)41 |
|  |  | MFG_L(R)76 |  |  | PCun_L(R)42 |
|  |  | MFG_L(R)77 |  |  | PCun_L(R)43 |
|  | IFG, Inferior Frontal Gyrus | IFG_L(R)61 |  |  | PCun_L(R)44 |
|  |  | IFG_L(R)62 |  | PoG, Postcentral Gyrus | PoG_L(R)41 |
|  |  | IFG_L(R)63 |  |  | PoG_L(R)42 |
|  |  | IFG_L(R)64 |  |  | PoG_L(R)43 |
|  |  | IFG_L(R)65 |  |  | PoG_L(R)44 |
|  |  | IFG_L(R)66 | **Insular Lobe** | INS, Insular Gyrus | INS_L(R)61 |
|  | OrG, Orbital Gyrus | OrG_L(R)61 |  |  | INS_L(R)62 |
|  |  | OrG_L(R)62 |  |  | INS_L(R)63 |
|  |  | OrG_L(R)63 |  |  | INS_L(R)64 |
|  |  | OrG_L(R)64 |  |  | INS_L(R)65 |
|  |  | OrG_L(R)65 |  |  | INS_L(R)66 |
|  |  | OrG_L(R)66 | **Limbic Lobe** | CG, Cingulate Gyrus | CG_L(R)71 |
|  | PrG, Precentral Gyrus | PrG_L(R)61 |  |  | CG_L(R)72 |
|  |  | PrG_L(R)62 |  |  | CG_L(R)73 |
|  |  | PrG_L(R)63 |  |  | CG_L(R)74 |
|  |  | PrG_L(R)64 |  |  | CG_L(R)75 |
|  |  | PrG_L(R)65 |  |  | CG_L(R)76 |
|  |  | PrG_L(R)66 |  |  | CG_L(R)77 |
|  | PCL, Paracentral Lobule | PCL_L(R)21 | **Occipital Lobe** | Cun, Cuneus Gyrus | Cun_L(R)51 |
|  |  | PCL_L(R)22 |  |  | Cun _L(R)52 |
| **Temporal Lobe** | STG, Superior Temporal Gyrus | STG_L(R)61 |  |  | Cun _L(R)53 |
|  |  | STG_L(R)62 |  |  | Cun _L(R)54 |
|  |  | STG_L(R)63 |  |  | Cun _L(R)55 |
|  |  | STG_L(R)64 |  | OcG, Occipital Gyrus | OcG_L(R)41 |
|  |  | STG_L(R)65 |  |  | OcG _L(R)42 |
|  |  | STG_L(R)66 |  |  | OcG _L(R)43 |
|  | MTG, Middle Temporal Gyrus | MTG_L(R)41 |  |  | OcG_L(R)44 |
|  |  | MTG_L(R)42 |  | sOcG, Superior Occipital Gyrus | sOcG _L(R)21 |
|  |  | MTG_L(R)43 |  |  | sOcG _L(R)22 |
|  |  | MTG_L(R)44 | **Subcortical Nuclei** | Amyg, Amygdala | Amyg_L(R)21 |
|  | ITG, Inferior Temporal Gyrus | ITG_L(R)71 |  |  | Amyg_L(R)22 |
|  |  | ITG_L(R)72 |  | Hipp, Hippocampus | Hipp_L(R)21 |
|  |  | ITG_L(R)73 |  |  | Hipp_L(R)22 |
|  |  | ITG_L(R)74 |  | Str, Striatum | Str_L(R)61 |
|  |  | ITG_L(R)75 |  |  | Str_L(R)62 |
|  |  | ITG_L(R)76 |  |  | Str_L(R)63 |
|  |  | ITG_L(R)77 |  |  | Str_L(R)64 |
|  | FuG, Fusiform Gyrus | FuG_L(R)31 |  |  | Str_L(R)65 |
|  |  | FuG_L(R)32 |  |  | Str_L(R)66 |
|  |  | FuG_L(R)33 |  | Tha, Thalamus | Tha_L(R)81 |
|  | PhG, Parahippocampal Gyrus | PhG_L(R)61 |  |  | Tha_L(R)82 |
|  |  | PhG_L(R)62 |  |  | Tha_L(R)83 |
|  |  | PhG_L(R)63 |  |  | Tha_L(R)84 |
|  |  | PhG_L(R)64 |  |  | Tha_L(R)85 |
|  |  | PhG_L(R)65 |  |  | Tha_L(R)86 |
|  |  | PhG_L(R)66 |  |  | Tha_L(R)87 |
|  | pSTS, posterior Superior Temporal Sulcus | pSTS_L(R)21 |  |  | Tha_L(R)88 |
|  |  | pSTS_L(R)22 |  | | |

| **Supplementary Table 3 Mathematical definitions of topological properties** | | |
| --- | --- | --- |
| **Topological properties** | **Binary and undirected definitions** | **Weighted and undirected definitions** |
| **Base concepts** | $N$ is the set of all nodes in the network, and $n$ is the number of nodes.  $L$ is the set of all links in the network, and $l$ is the number of links.  $(i,j)$ is a link between $i$ and $j (i,j\in N)$.  $a_{ij}$ is the connection status between *i* and *j*: $a_{ij}$ = 1 when link $(i,j)$ exists; $a_{ij}$ = 0 otherwise.  We compute the number of links as $l$ = $\sum_{i,j\in N} a_{ij}$. | $N$ is the set of all nodes in the network, and $n$ is the number of nodes.  $L$ is the set of all links in the network, and $l$ is the number of links.  Links $(i,j)$ are associated with connection weights $w_{ij}.0\leq w_{ij}\leq1$ for all $i$ and $j$.  $l^{w}$ is the sum of all weights in the network, computed as $l^{w}=\sum_{i,j\in N} w_{ij}$. |
| **Degree centrality** | Degree of a node 𝑖, $k_{i}=\sum_{j\in N} a_{ij}$ | Weighted degree of $i$, $k_{i}^{w}=\sum_{i,j\in N} w_{ij}$ |
| **Shortest path length** | Shorted path length between $i$ and $j$,  $d_{ij}=\sum_{a_{uv}\in g_{i\to j}} a_{uv}$ ,  where $g_{i\to j}$ is the shortest path between $i$ and $j$. | Shorted weighted path length between $i$ and $j$, $d_{ij}^{w}=\sum_{a_{uv}\in g_{i\to j}^{w}} f\left( w_{uv} \right)$ ,  where $f$ is a map from weight to length and $g_{i\to j}^{w}$ is the shortest weighted path between $i$ and $j$. |
| **Characteristic path length** | Characteristic path length of the network, $L=1/n\times\sum_{i\in N} {\sum_{j\in N,j\neq i} d_{ij}}/{(n-1)}$ , where $L_{i}$ is the average distance between node *i* and all other nodes. | Weighted characteristic path length,  $L^{w}=1/n\times\sum_{i\in N} {\sum_{j\in N,j\neq i} d_{ij}^{w}}/{(n-1)}$,  $d_{ij}^{w}$ is the shorted weighted path length between *i* and *j.* |
| **Global efficiency** | Binary global efficiency,  $E=1/n\times\sum_{i\in N} {\sum_{j\in N,j\neq i} {(d_{ij})}^{-1}}/{(n-1)}$ | Weighted global efficiency,  $E^{w}=1/n\times\sum_{i\in N} {\sum_{j\in N,j\neq i} {{(d}_{ij}^{w})}^{-1}}/{(n-1)}$ |
| **Clustering coefficient** | Binary clustering coefficient,  $C=1/n\times\sum_{i\in N} {{2t}_{i}}/{k_{i}(k_{i}-1)}$.  $t_{i}=1/2{\sum_{h,j\in N} {(a}_{hj}a_{ij}a_{ih})}$*,*  $t_{i}$ is number of triangles around a node *i*. | Weighted clustering coefficient,  $C^{w}=1/n\times\sum_{i\in N} {{2t}_{i}^{w}}/{k_{i}(k_{i}-1)}$.  $t_{i}^{w}=1/2{\sum_{h,j\in N} {(w}_{hj}w_{ij}w_{ih})}^{1/3}$  $t_{i}^{w}$ is weighted geometric mean of triangles around *i*. |
| **Small-worldness** | Binary network small-worldness,  $S=C/C_{rand}$ / $L/L_{rand}$ | Weighted network small-worldness,  $S^{w}=C^{w}/C_{rand}^{w}$ / $L^{w}/L_{rand}^{w}$ |
| **Local efficiency** | Binary local efficiency.$E_{loc}=1/n\sum_{i\in N} \sum_{j,h\in N,j\neq i} \left( a_{ij}a_{jh}\left[ d_{jh}\left( N_{i} \right)^{-1} \right] \right)/k_{i}(k_{i}-1)$ | Weighted local efficiency.$E_{loc}^{w}=1/n\sum_{i\in N} \sum_{j,h\in N,j\neq i} \left( w_{ij}w_{ih}\left[ d_{jh}^{w}\left( N_{i} \right)^{-1} \right] \right)^{\frac{1}{3}}/k_{i}(k_{i}-1)$ |
| **Betweenness centrality** | Betweenness centrality of node *i*,  $b_{i}=1/(n-1)(n-2)\times{\sum_{h,j\in N,j\neq i,j\neq h,h\neq i} p_{hi}(i)}/{p_{hi}}$ , where $p_{hi}$ is the number of shortest paths between *h* and *j*. | Betweenness centrality is computed equivalently on weighted and directed networks, provided that path lengths are computed on respective weighted or directed paths. |

| **Supplemental Table 4 Connection properties of the rich-club organization** | | | | | | | |
| --- | --- | --- | --- | --- | --- | --- | --- |
|  |  |  |  |  |  |  |  |
| **Connection properties** | | **HC** | **MCI-AD** | **WMH-NC** | **WMH-MCI** | ***p*** value | |
|  |  |  |  |  |  | **HC vs MCI-AD** | **HC vs WMH-NC vs  WMH-MCI** |
| **strength** | RC | 83.41±27.66 | 67.71±22.85 | 69.91±18.81 | 72.60±21.23 | <0.001 | 0.001 |
|  | FC | 514.93±58.20 | 486.63±53.24 | 492.77±42.78 | 496.44±53.56 | 0.002 | 0.004 |
|  | LC | 873.52±81.56 | 916.97±73.71 | 907.57±58.79 | 901.59±70.68 | 0.001 | 0.002 |
| **degree** | RC | 85.67±28.29 | 69.59±23.39 | 71.97±19.34 | 74.63±21.74 | <0.001 | 0.001 |
|  | FC | 529.54±59.23 | 500.59±54.48 | 507.35±43.85 | 510.71±54.49 | 0.002 | 0.005 |
|  | LC | 898.87±84.68 | 943.64±76.06 | 934.87±60.35 | 928.17±73.90 | 0.001 | 0.002 |
| Abbreviation: HC, health control; NC, normal cognition; MCI-AD, mild cognitive impairment due to Alzheimer's disease; WMH, white matter hyperintensities; RC, rich-club connections; FC, feeder connections; LC, local connections. | | | | | | | |

**Supplemental Figure 1 Global properties of the susceptibility network**


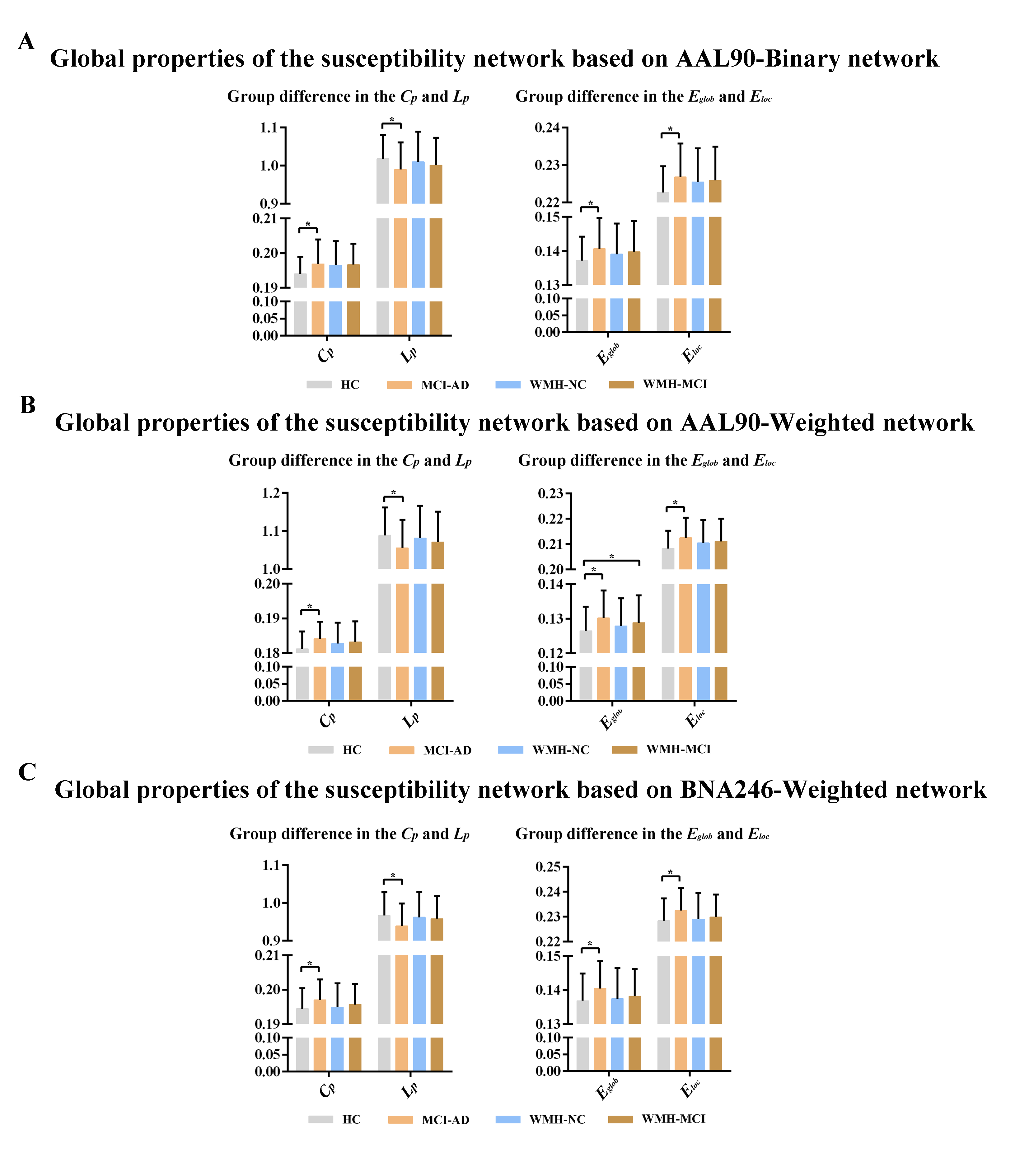


**Supplemental Figure 2 Regional properties of the susceptibility network**


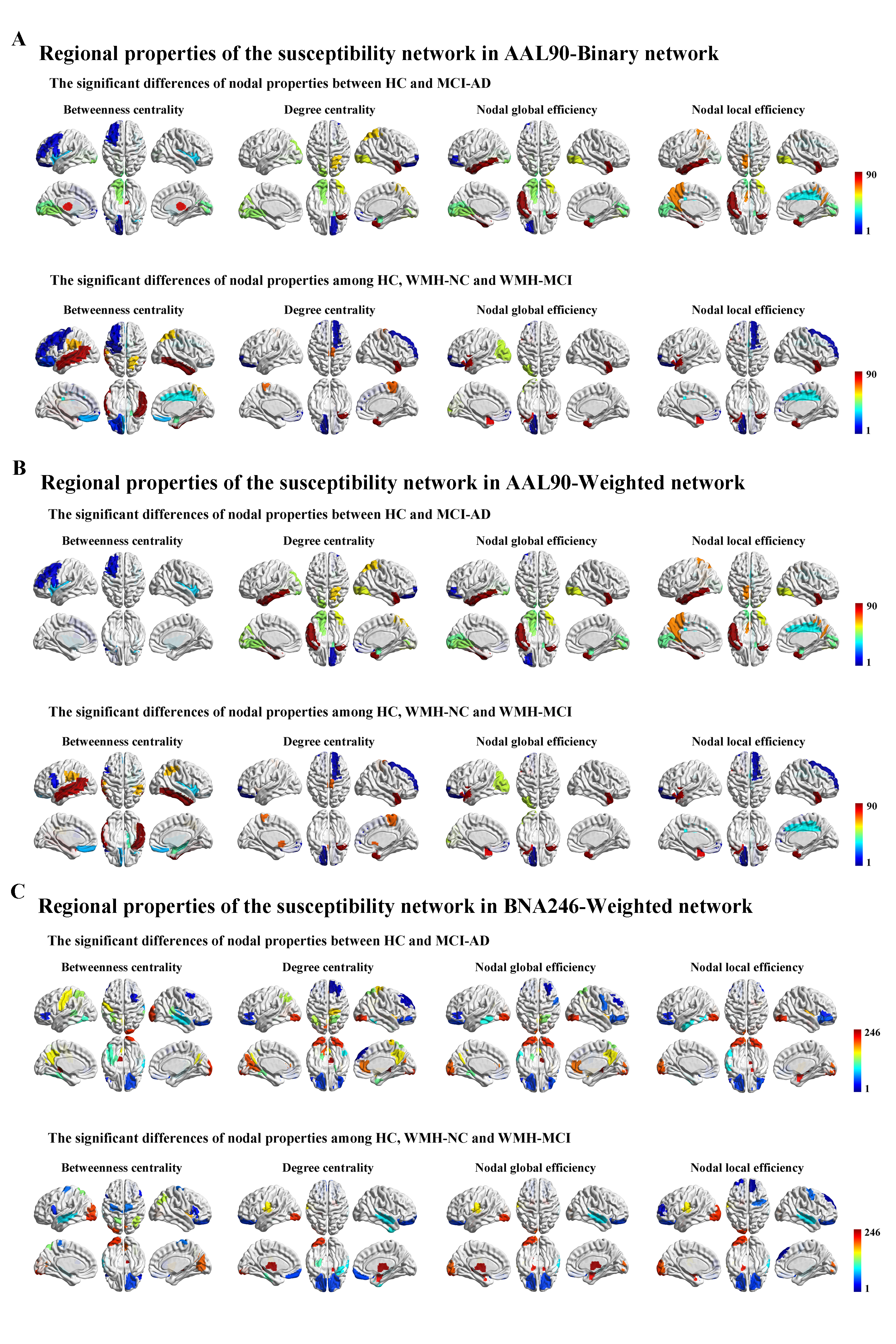


**Supplemental Figure 3 Rich-club organization analysis based on AAL90-Binary network**


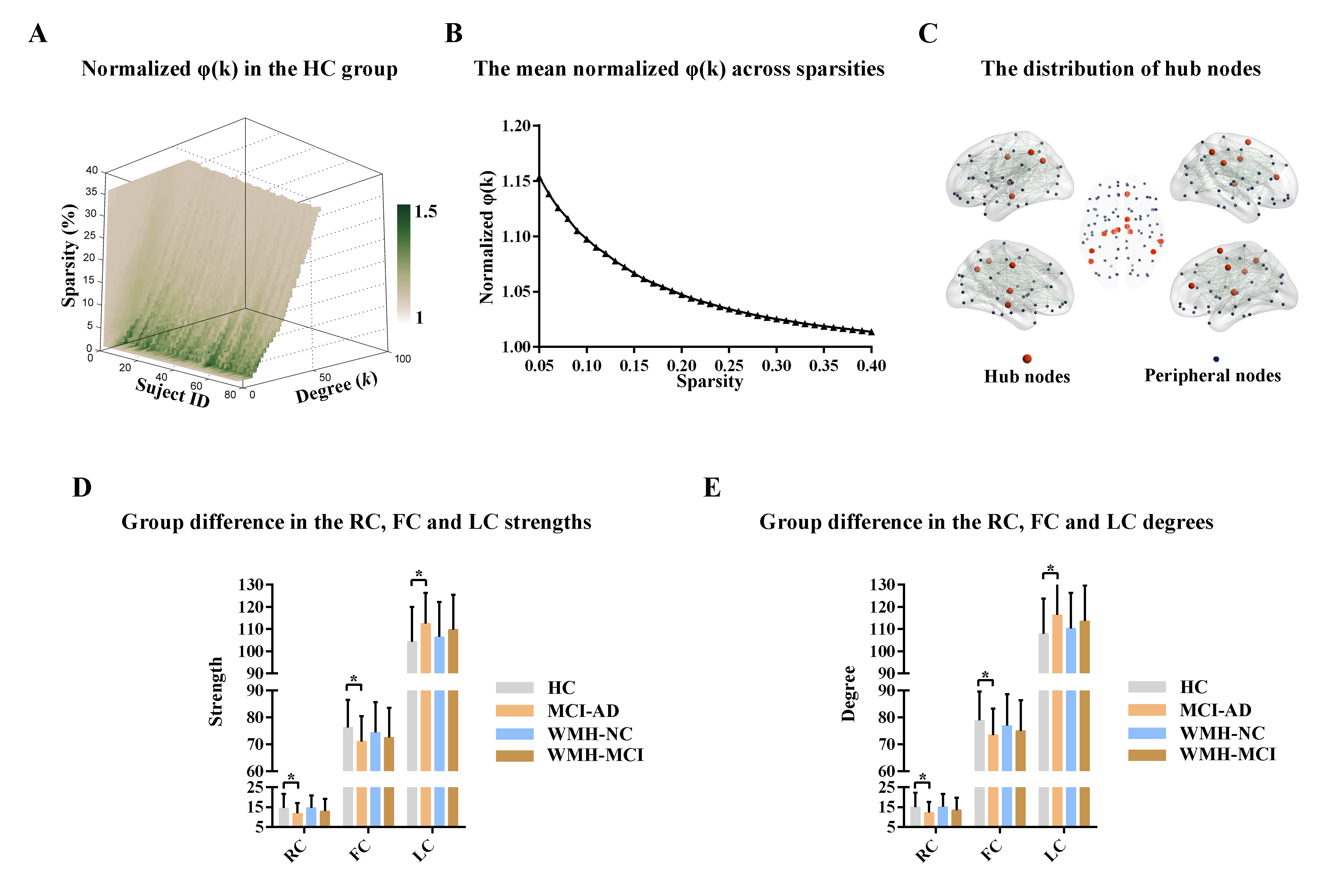


**Supplemental Figure 4 Rich-club organization analysis based on AAL90-Weighted network**


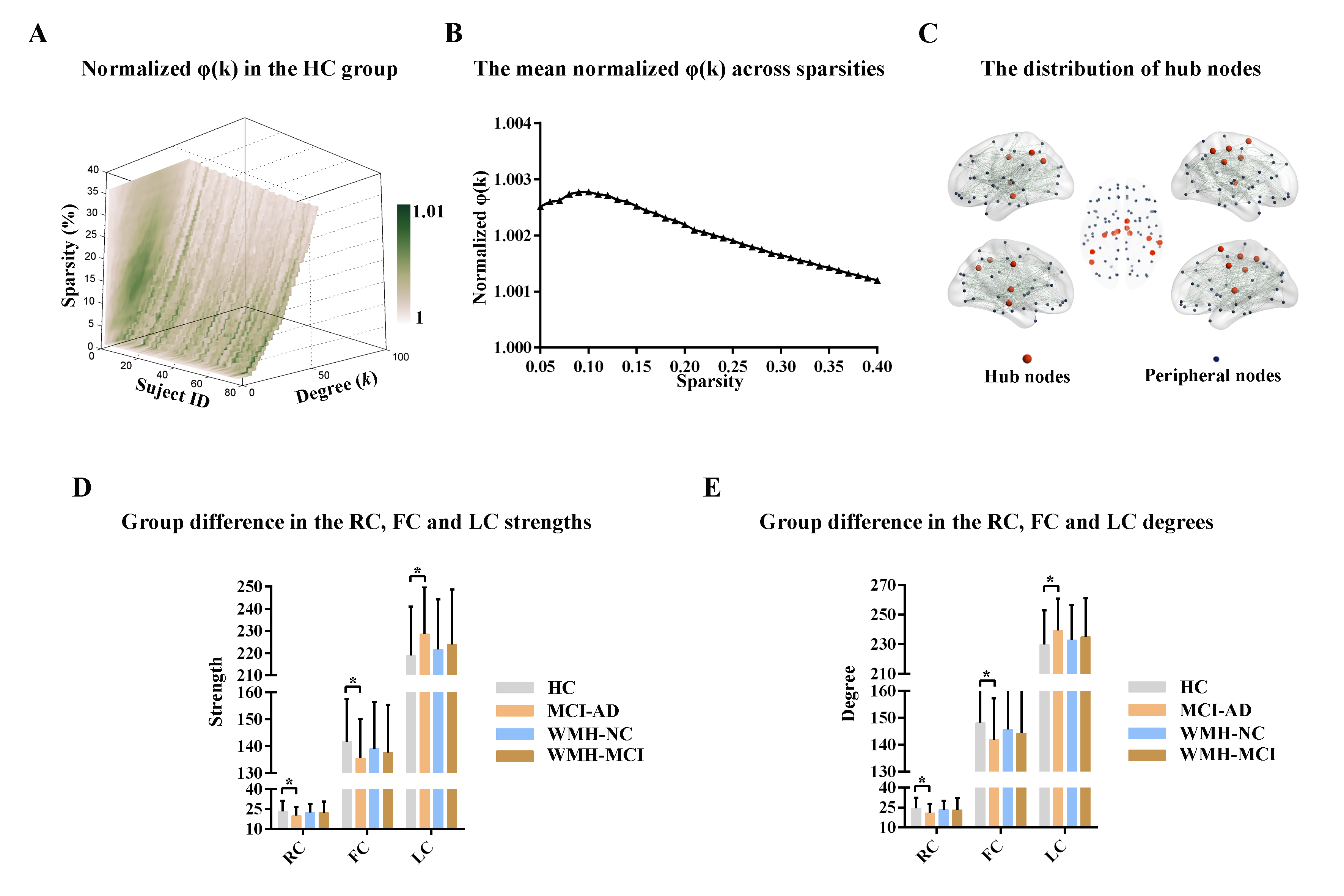


**Supplemental Figure 5 Rich-club organization analysis based on BNA246-Weighted network**


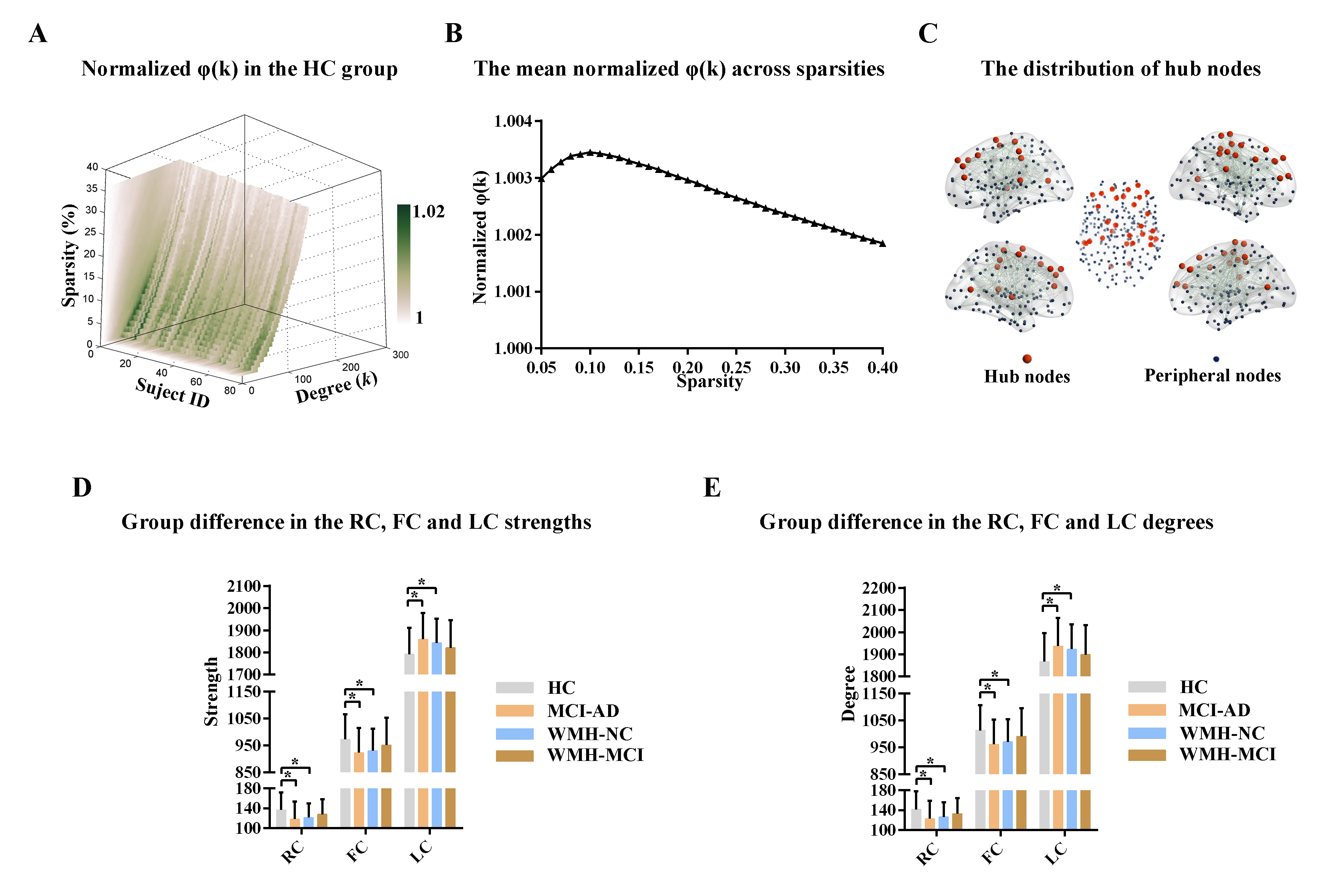

Supplement: Supplementary file 1 — Supplementary materials [file 41398_2024_2861_MOESM1_ESM.docx]
